# VirA+EmiC project: Evaluating real‐world effectiveness and sustainability of integrated routine opportunistic hepatitis B and C testing in a large urban emergency department

**DOI:** 10.1111/jvh.13676

**Published:** 2022-04-09

**Authors:** Gaia Nebbia, Murad Ruf, Laura Hunter, Sooria Balasegaram, Terry Wong, Ranjababu Kulasegaram, Julian Surey, Zana Khan, Jack Williams, Basel Karo, Luke Snell, Barnaby Flower, Hannah Evans, Sam Douthwaite

**Affiliations:** ^1^ 8945 Department of Infection Guy's and St Thomas' NHS Foundation Trust London UK; ^2^ Gilead Sciences Ltd UK & Ireland Medical Department London UK; ^3^ 8945 Department of Emergency Medicine Guy's and St Thomas' NHS Foundation Trust London UK; ^4^ Field Epidemiology Service National Infection Service UK Health Security Agency, London UK London UK; ^5^ 8945 Gastroenterology and Hepatology Department Guy's and St Thomas' NHS Foundation Trust London UK; ^6^ 8945 Department of HIV/GU Medicine Guy's and St Thomas' NHS Foundation Trust London UK; ^7^ Institute for Global Health University College London London UK; ^8^ Universidad Autónoma de Madrid Madrid Spain; ^9^ Institute of Epidemiology and Health Care UCL and South London and Maudsley Trust London UK; ^10^ Department of Health Services Research and Policy London School of Hygiene and Tropical Medicine London UK; ^11^ International Health Protection (ZIG 1) Robert Koch Institute (RKI) Berlin Germany; ^12^ Division of Infectious Diseases Imperial College London London UK; ^13^ UK Field Epidemiology Training Programme UK Health Security Agency London UK

**Keywords:** electronic health records, emergency department, hepatitis B, hepatitis C, hospital

## Abstract

Innovative testing approaches and care pathways are required to meet global hepatitis B virus (HBV) and hepatitis C virus (HCV) elimination goals. Routine blood‐borne virus (BBV) testing in emergency departments (EDs) in high‐prevalence areas is suggested by the European Centre for Disease Prevention and Control (ECDC) but there is limited evidence for this. Universal HIV testing in our ED according to UK guidance has been operational since 2015. We conducted a real‐world service evaluation of a modified electronic patient record (EPR) system to include opportunistic opt‐out HBV/reflex‐HCV tests for any routine blood test orders for ED attendees aged ≥16 years. Reactive laboratory results were communicated directly to specialist clinical teams. Our model for contacting patients requiring linkage to care (new diagnoses/known but disengaged) evolved from initially primarily hospital‐led to collaborating with regional health and community service networks. Over 11 months, 81,088 patients attended the ED; 36,865 (45.5%) had a blood test. Overall uptake for both HBV and HCV testing was 75%. Seroprevalence was 0.9% for hepatitis B surface antigen (HBsAg) and 0.9% for HCV antigen (HCV‐Ag). 79% of 140 successfully contacted HBsAg+patients required linkage to care, of which 87% engaged. 76% of 130 contactable HCV‐Ag+patients required linkage, 52% engaged. Our results demonstrate effectiveness and sustainability of universal ED EPR opt‐out HBV/HCV testing combined with comprehensive linkage to care pathways, allowing care provision particularly for marginalized at‐risk groups with limited healthcare access. The findings support the ECDC BBV testing guidance and may inform future UK hepatitis testing guidance.

**Abbreviations**
BBV: blood‐borne virus
CI: confidence interval
ECDC: European Centre for Disease Prevention and Control
ED: emergency department
EPR: electronic patient record
GP: general practitioner
HBsAg: hepatitis B surface antigen
HBV: hepatitis B virus
HCV: hepatitis C virus
HCV‐Ab: HCV antibody
HCV‐Ag: HCV antigen
HIV: human immunodeficiency virus
Ig: immunoglobulin
IQR: interquartile range
NHS: National Health Service
NICE: National Institute for Health and Care Excellence
PHE: Public Health England
PR: prevalence ratio

## 1 INTRODUCTION

Hepatitis B virus (HBV) and hepatitis C virus (HCV) infections are a considerable cause of morbidity and mortality worldwide and in the UK: recent Public Health England (PHE) estimates suggest that, in 2019, approximately 118,000 people in the UK were living with chronic HCV infection.^1^ For HBV, no PHE estimates are available; the Polaris Observatory estimated that, in 2016, approximately 441,000 people were living with chronic HBV in the UK.^2^

There is now broad access to efficacious treatments that reduce both mortality and morbidity for both viral infections,^3^, ^4^ yet key challenges remain.^5^ This is primarily because many infections remain undiagnosed: in 2018, around two‐thirds of chronic HCV infections were estimated to be undiagnosed.^6^ 81% of HBV infections in the UK were estimated to be undiagnosed in 2016.^2^

The 2016 World Health Organization (WHO) Global Health Sector Strategy on Viral Hepatitis prioritized the need for innovative testing strategies and efficient linkage to care pathways with the aim of eliminating viral hepatitis as a major public health threat by 2030.^5^

Routine blood‐borne virus (BBV) testing in emergency departments (EDs) in high‐prevalence areas is suggested by the European Centre for Disease Prevention and Control (ECDC) but with concession that evidence for its effectiveness is lacking.^7^ In England, there were >24.8 million attendances at EDs in 2018–2019.^8^ For often marginalized populations at high risk of BBVs, the ED is often a key healthcare access point.^9^, ^10^ Findings from pilot ED opt‐out viral testing studies and recent multicentre UK ED seroprevalence surveys^11^, ^12^ suggested a consistent, high level of active undiagnosed BBV infections, much higher than at general population level.^13^, ^14^ UK guidance from the National Institute for Health and Care Excellence (NICE) recommends that, in high‐prevalence areas, human immunodeficiency virus (HIV) testing is universally offered to adult ED attendees who are undergoing blood tests for another healthcare reason.^15^ However, in contrast, no similar consideration exists in current UK hepatitis testing guidelines.^16^

In 2014, a 1‐week opt‐out ED testing campaign in nine UK EDs showed that this type of testing was feasible and effective.^17^ Subsequently, pilot initiatives in London (UK),^18^, ^19^ and Dublin (Ireland)^20^ have shown success using opt‐out ED blood screening for HBV and HCV in identifying undiagnosed patients or those lost to follow‐up and linking them to care. Previously we evaluated a 6‐week pilot study in our centre, which demonstrated success of our model in a large inner‐city ED serving a diverse population with high levels of social deprivation.^21^ This current paper describes an extension of the model to evaluate its real‐world sustainability. In particular, we discuss the challenges that were faced when integrating sustainable effective linkage to care into clinical routine, and methods that we employed to overcome these challenges.

## 2 METHODS

### 2.1 Study design, setting and participants

We conducted a clinical service development evaluation at the ED of Guy's and St Thomas’ NHS Foundation Trust in London, UK. The project was conducted in two phases: phase 1 lasted 5 months (October 2016–February 2017) and phase 2 lasted 6 months (December 2017–May 2018). In contrast to traditional healthcare professional‐initiated testing models, the electronic patient record (EPR) system was modified to pre‐select HBV and HCV tests for any blood test orders in the ED for patients ≥16 years on an opt‐out basis, unless a positive viral hepatitis test result within the previous 6 months was available on the EPR system. Multilingual information leaflets were handed out to patients at registration and provided by the clinician, posters were also available throughout the ED. These leaflets included disease awareness, information on testing policy and process, and how to access care for either infection. Additionally, all eligible patients were verbally informed before blood draw that viral hepatitis tests would be carried out unless they declined (opted‐out). Staff in the ED department were regularly trained by senior clinicians. This project was a service development evaluation (http://www.hra‐decisiontools.org.uk/research/) and did not require further ethical review by an NHS Ethics Committee or management permission through NHS Research & Development.

### 2.2 Laboratory methods

Laboratory testing was carried out according to routine local protocol. Current HBV infection was diagnosed upon detection of hepatitis B surface antigen (HBsAg) (ARCHITECT; Abbott Laboratories). If HBsAg was reactive on screening assay, specificity was confirmed with a neutralization step, and reflex testing of hepatitis B core immunoglobulin (Ig)G, IgM antibodies and e‐markers. HCV testing comprised an initial antibody screening test (HCV‐Ab) (ARCHITECT; Abbott Laboratories), followed by same‐sample reflex‐HCV antigen (HCV‐Ag) (ARCHITECT; Abbott Laboratories) testing for positive or equivocal initial HCV‐Ab results. For negative or equivocal HCV‐Ag tests, a second HCV‐Ab test (Bio‐Rad Inc.) was performed to confirm the presence of HCV antibodies detected in the initial screening assay. Whenever the HCV‐Ab or HBsAg result had been previously demonstrated using this algorithm, any subsequent sample from the same individual was only tested using the screening test and reported as a confirmed result. HIV testing as an opt‐out test for the ED attendees had already integrated into routine ED testing practice in our hospital since 2015; this is reported separately^22^ and, therefore, data regarding HIV testing outcome were not reported for this service evaluation.

### 2.3 Follow‐up/linkage to care

Patients testing positive for HBsAg and HCV‐Ag had their EPR checked by the study investigators. If there was documentation indicating that the patient's result reflected a known diagnosis who were already engaged in care, no further action was taken. Patients who were HCV‐Ab positive but HCV‐Ag negative (and for whom no prior local HCV RNA status was found) were asked by letter to attend their general practitioner (GP) for a follow‐up HCV RNA sample to exclude current infection. As a linkage to care pathway already existed for patients with HIV infection, no further action was taken for patients with dual infection with HIV and either HBV or HCV.

Patients with a confirmed infection who were still alive at point of review were considered eligible for contact (see below). Among patients who were informed of their diagnosis (successfully contacted), those who were either newly diagnosed or known but disengaged with care, or those whose status had not been clarified (unknown) during the initial contact, were classed as ‘requiring linkage to care’.

If the patient attended at least one appointment, the outcome of the linkage to care was recorded as ‘engaged with care’. During the initial appointment, patients underwent assessment in accordance with the current UK clinical guidance.^4^, ^23^ Patients who were lost to follow‐up at each stage of the linkage pathway were described. Treatments offered for HCV and HBV were in accordance with the NICE and NHS England guidelines.^4^, ^23^

### 2.4 Evolution of contacting and engaging patients during project

Linkage to care approaches were under constant review and evolved throughout the service evaluation. At the beginning of the project, the linkage to care pathway included a direct telephone call to the patient informing them of diagnosis, care status assessment and offering specialist clinical appointments. While effective, the initial consultant‐led model employed during the 6‐week pilot^21^ proved unsustainable during this evaluation owing to the complexity of identifying valid contact details in this often marginalized patient cohort.

Therefore, a dedicated part‐time research nurse was employed as care navigator to lead contacting of the patient firstly by telephone (two attempts 24 h apart) and then, if unsuccessful, by a text message to the mobile number on record. If the patient had a valid address, a letter was also posted. If the patient was registered with GP, the research nurse notified the GP by post, and recorded all data in the project database. In addition, electronic notes were left on hospital and ED re‐attendance patient records. Hospital homeless teams checked community health records for contact details or GP details. For patients who could not be contacted despite these efforts, their information was shared (in accordance with national information governance policies) with local hospital homeless teams and (where relevant) the Find and Treat team (UCLH NHS Trust), a dedicated pan‐London NHS community inclusion health outreach team.^24^ NHS Find and Treat uses peer‐support coupled with bespoke pan‐London homeless databases and links with shelters and charities to identify, contact and engage patients with care.

### 2.5 Data collection and management

A dataset that included all ED attendees aged ≥16 years, those with routine blood tests and those who were tested for HBsAg and/or HCV‐Ab during the study period was extracted from the EPR system. This dataset included demographic information, including age, sex, ethnicity and residence type (including hostel, no fixed abode). For patients testing positive for HBsAg and/or HCV‐Ag, a separate linkage to care dataset was also compiled, which included contact, diagnosis and linkage to care outcomes. Each patient was assigned a unique patient identifier so that datasets could be collated and deduplicated. Data were handled in accordance with information governance policies to ensure patient confidentiality. Only staff with direct patient care responsibilities had access to patient‐identifiable data. Equivocal test results for HBsAg or HCV‐Ab with a negative HCV‐Ag result were recorded as negative. Unsuitable samples or samples that were not received in the laboratory were recorded as ‘not tested'. For patients who attended the ED multiple times during the study period, the earliest record was retained and updated with additional information from subsequent records, unless there was a later attendance linked with a positive test result. HIV status was recorded as negative, positive or unknown. Age was recorded as a categorical variable (16–29, 30–49, 50–69, ≥70 years) and ethnicity was classified into six categories, in line with UK census categories. ED arrival was categorized as day (08:00–19:59) or night (20:00–07:59) and also by arrival day (weekday: Monday to Friday, weekend: Saturday or Sunday).

### 2.6 Statistical analysis

Continuous variables were described using medians with interquartile ranges (IQRs). Categorical variables were described as absolute and relative frequencies. Overall and stratified seroprevalence estimates with their 95% CI were calculated for the HCV‐Ag and HBsAg tests. To investigate factors associated with requiring a HCV or HBV test and for being seropositive, we applied univariable and multivariable Poisson regression models to calculate prevalence ratios (PRs). All tests were two sided with 95% CI; the level of significance was set at *p* < 0.05. All analyses were performed using STATA version 15 software (StataCorp LP).

## 3 RESULTS

**FIGURE 1:**
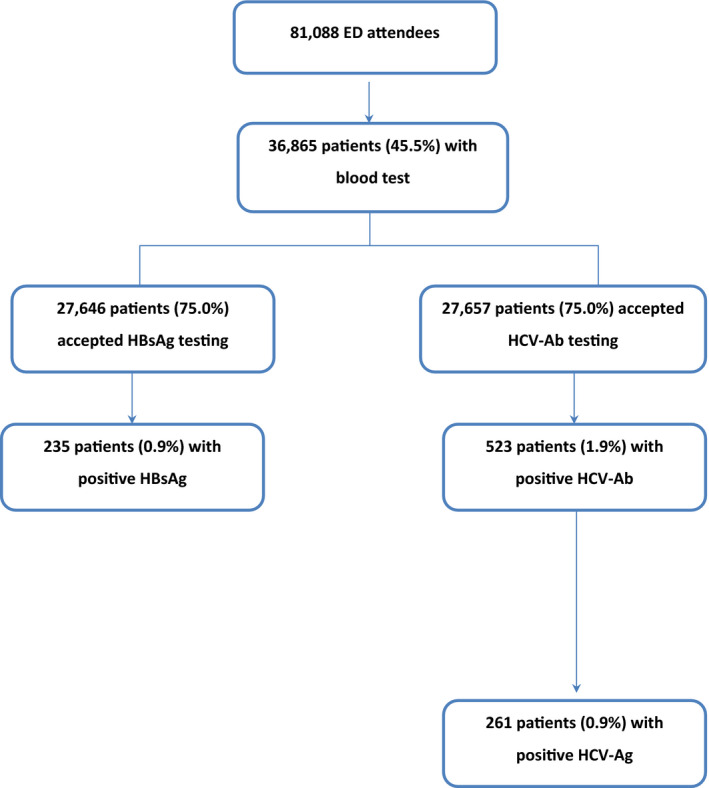
Patients included in the service evaluation. Abbreviations: ED, emergency department; HBsAg, hepatitis B surface antigen; HCV‐Ab, hepatitis C virus antibody; HCV‐Ag, hepatitis C virus antigen

During the 11‐month service evaluation period, 81,088 patients attended the ED and, of those, 36,865 (45.5%) received a blood test. There was an approximately equal distribution of female to male ED participants (51% female); 37% were of White British ethnicity, 19% White other and 18% Black/Black British. The median (IQR) age was 44 (30–61) years. Figure 1 shows the numbers and proportions of patients prospectively screened who received the HBsAg, HCV‐Ab and HCV‐Ag tests, and the numbers and proportions of patients testing positive for each.

### 3.1 Hepatitis B

#### 3.1.1 Uptake of hepatitis B testing

Overall uptake of HBsAg testing was 75.0% (27,646/36,865). Testing uptake was relatively constant during the two phases of the evaluation (Figure S1). Test uptake was consistently >72% (range 72.6–77.3%) across strata (sex, age, ethnicity, homeless status, ED arrival day and ED arrival time).

#### 3.1.2 Prevalence and risk factors for seropositivity

Of 27,646 patients tested for HBsAg, 235 patients tested positive, giving an overall seroprevalence of 0.9% (95% CI 0.8–1.0; Table 1). With univariable testing (Table S1), factors significantly associated (*p* < 0.001) with testing positive for HBsAg were being male (vs female; PR 1.5, 95% CI 1.2–2.0), aged 30–49 years (vs aged 16–29 years; PR 3.4, 95% CI 2.2–5.3) or 50–69 years (vs aged 16–29 years; PR 4.0, 95% CI 2.5–6.3), ethnicity other than White British (vs White British; PR >6.6) and positive HIV status (vs negative; PR 6.0, 95% CI 2.9–12.8). Arriving at the ED at night was negatively associated with testing positive for HBsAg (vs day arrival; PR 0.7, 95% CI 0.6–1.0, *p* = 0.004). With multivariable testing (Table S1), all of the above‐mentioned factors associated with testing positive for HBsAg remained statistically significant. Seropositivity according to study period and age group are shown graphically in Figure S1.

**TABLE 1:** Seroprevalence of HBsAg

Characteristics	Tested	Positive HBsAg	Seroprevalence of HBsAg, % (95% CI)
Total	27,646	235	0.9 (0.8–1.0)
Sex			
Female	13,939	94	0.7 (0.6–0.8)
Male	13,707	141	1.0 (0.9–1.2)
Age group, years			
16–29	6,928	23	0.3 (0.2–0.5)
30–49	9,437	106	1.1 (0.9–1.1)
50–69	6,851	91	1.3 (1.1–1.6)
≥70	4,430	15	0.3 (0.2–0.6)
Ethnicity			
White British	10,271	12	0.1 (0.1–0.2)
White other	5,223	40	0.8 (0.6–1.0)
Black/Black British	4,927	100	2.0 (1.7–2.5)
Asian	1,693	34	2.0 (1.4–2.8)
Mixed/other	1,869	16	0.9 (0.5–1.4)
Not recorded	3,663	33	0.9 (0.6–1.3)
Homeless			
No	25,490	209	0.8 (0.7–0.9)
Yes	663	10	1.5 (0.8–2.8)
ED arrival day			
Weekday	20,688	180	0.9 (0.8–1.0)
Weekend	6,723	55	0.8 (0.6–1.1)
ED arrival time			
Day (08:00–19:59)	18,208	170	0.9 (0.8–1.1)
Night (20:00–07:59)	9,438	65	0.7 (0.5–0.9)

Note

Data are from Guy's and St Thomas' NHS Foundation Trust, London (2016–2018).

Abbreviations: CI, confidence interval; ED, emergency department; HBsAg, hepatitis B surface antigen; NHS, National Health Service.

##### Hepatitis B: linkage to care

**FIGURE 2:**
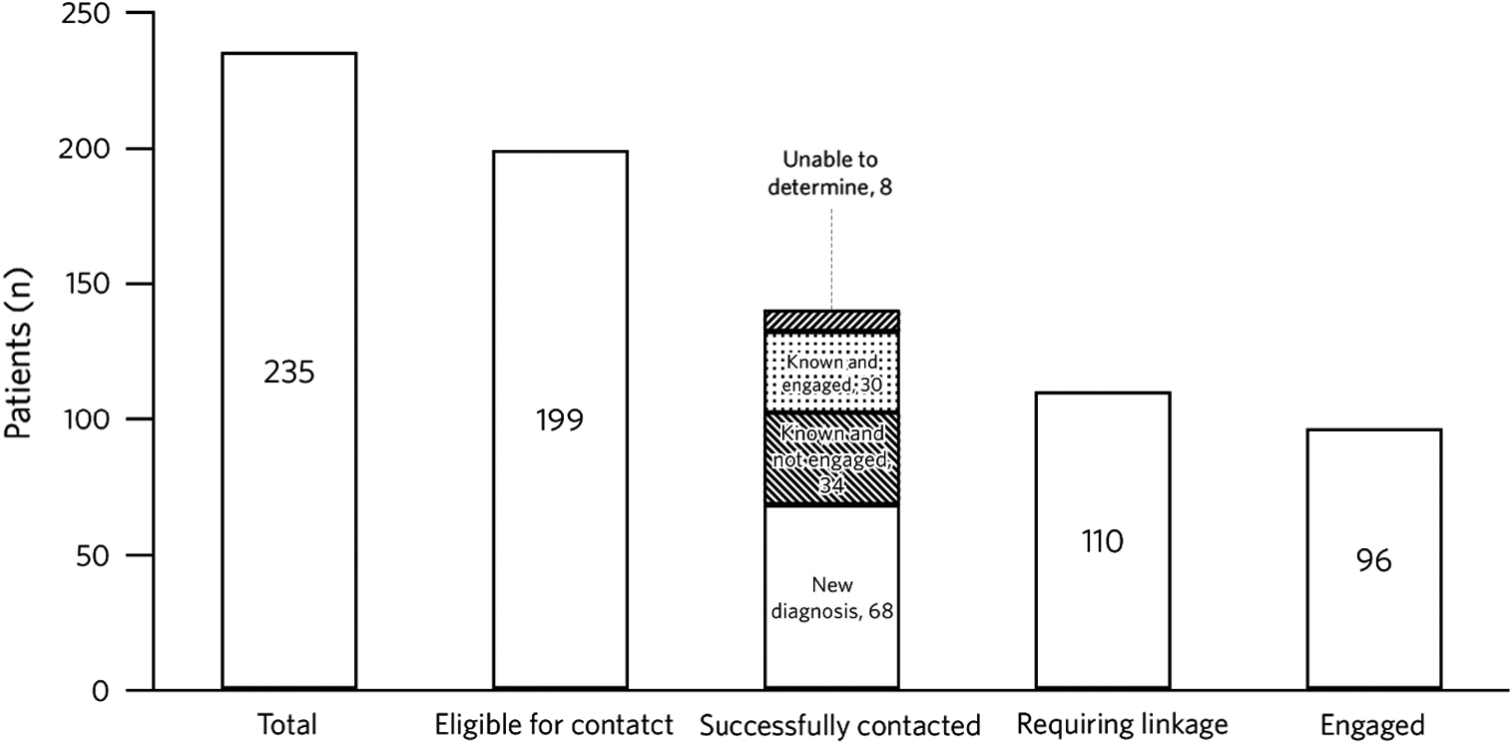
Linkage to care in patients with positive HBsAg status. Abbreviation: HBsAg, hepatitis B surface antigen

Among the 235 patients who tested positive for HBsAg, 36 (15%) did not need any further follow‐up as local care records showed that they were already engaged in care at time of testing, had a known end‐of‐life diagnosis (unrelated to hepatitis B) or died shortly after testing (Figure 2). Of the remaining 199 patients eligible for contact, 140 patients (70%) received their hepatitis B status, but we were unable to confirm contact in 59 patients (30%). Of the 140 patients with successful contact, 68/140 (48%) were new diagnoses, 64/140 (46%) were known diagnoses, of which 34/64 (53%) were not engaged at the time of testing and 8/140 (6%) patients were defined as ‘unknown’ as we were unable to gain information regarding their knowledge of the diagnosis and/or engagement into care at time of contact. Of the 110 patients requiring linkage to care (new or disengaged), 96/110 (87%) were successfully linked (of which 86/110 (80%) were linked locally and 10/110 (9%) elsewhere, as confirmed by follow‐up with local centres). Of the 86 patients who attended locally, 46/86 (53%) attended more than one clinic visit at time of data review. Of linked patients, 41/96 (43%) indicated that they were born outside the UK. All engaged patients had a fixed abode.

### 3.2 Hepatitis C

#### 3.2.1 Uptake of hepatitis C testing

Uptake of HCV‐Ab testing was 75.0% (27,657/36,865) (Figure 1). Testing uptake was relatively constant during the two phases of the evaluation and consistently high across age groups (Figure S2). Uptake was >72% (range 72.5–77.5%) across all demographic strata, regardless of sex, age, ethnicity, homeless status, ED arrival day and ED arrival time.

#### 3.2.2 Prevalence and risk factors for seropositivity identified using routine ED screening

Of 27,657 patients tested for HCV‐Ab, 523 patients tested positive, corresponding to an HCV‐Ab seroprevalence of 1.9% (95% CI 0.7–2.0). Of the 457 HCV‐Ab‐positive results for which a reflex‐HCV‐Ag test was performed, 261 patients tested positive for HCV‐Ag, corresponding to a seroprevalence for chronic HCV infection of 0.9% (95% CI 0.8–1.0) (Table 2). High seroprevalence rates of HCV‐Ag were observed among male patients (1.6%, 95% CI 1.4–1.8) and those of White British ethnic background (1.3%, 95% CI 1.0–1.5).

**TABLE 2:** Seroprevalence of HCV‐Ag

Characteristics	Tested	Positive HCV‐Ag	Seroprevalence HCV‐Ag, % (95% CI)
Sex			
Female	13,993	49	0.4 (0.3–0.5)
Male	13,664	212	1.6 (1.4–1.8)
Age group, years			
16–29	6,964	22	0.3 (0.2–0.5)
30–49	9,464	142	1.5 (1.3–1.8)
50–69	6,803	83	1.2 (1.0–1.5)
≥70	4,426	14	0.3 (0.2–0.5)
Ethnicity			
White British	10,247	132	1.3 (1.0–1.5)
White other	5,234	53	1.0 (0.8–1.3)
Black/Black British	4,932	17	0.3 (0.2–0.5)
Asian	1,695	5	0.3 (0.1–0.7)
Mixed/other	1,884	17	0.9 (0.6–1.4)
Not recorded	3,665	37	1.0 (0.7–1.4)
Homeless			
No	25,496	139	0.5 (0.5–0.6)
Yes	668	96	14.7 (12.2–17.6)
ED arrival day			
Weekday	20,875	194	0.9 (0.8–1.1)
Weekend	6,782	67	1.0 (0.8–1.3)
ED arrival time			
Day (08:00–19:59)	18,200	147	0.8 (0.7–0.9)
Night (20:00–07:59)	9,457	114	1.2 (0.8–1.1)

Note

Data are from Guy's and St Thomas' NHS Foundation Trust, London (2016–2018).

Abbreviations: CI, confidence interval; ED, emergency department; HCV‐Ag, hepatitis C virus antigen; NHS, National Health Service.

Seroprevalence of HCV‐Ag was highest among homeless people (14.7%) and HIV‐positive individuals (7.2%). In multivariable regression models (Table S2), factors significantly associated (*p* < 0.01) with testing positive for HCV‐Ag were being male (vs female; PR 2.5, 95% CI 1.7–3.5), being homeless (vs having a fixed abode; PR 16.6, 95% CI 12.5–22.1) and having HIV‐positive status (vs negative; PR 2.8, 95% CI 1.5–5.1, respectively). Compared with patients aged 16–29 years, patients aged 30–49 years and 50–69 years were at significantly higher risk for testing positive for HCV‐Ag (PR 3.6, 95% CI 2.2–6.0, *p* < 0.01 and PR 3.2, 95% CI 1.9–5.4, *p* < 0.01, respectively) (Table 2). Seropositivity according to age group is shown graphically in Figure S2. All ethnic groups were at significantly lower risk for HCV infection compared with patients of White British ethnicity.

#### 3.2.3 Hepatitis C linkage to care

In addition to the 261 patients who tested positive for HCV‐Ag in the ED testing programme, a further 12 patients tested positive for HCV‐Ab but did not have an HCV‐Ag test carried out at this point because of previous HCV‐Ag positivity (see laboratory methods). However, these patients were considered eligible for contact following review of the result in their EPR, which indicated current HCV infection. Of these 273 patients, 22 were found to be co‐infected with HIV and their follow‐up was devolved to the HIV team and, therefore, not included in our analysis. This left 251 patients included in the linkage to care analysis.

**FIGURE 3:**
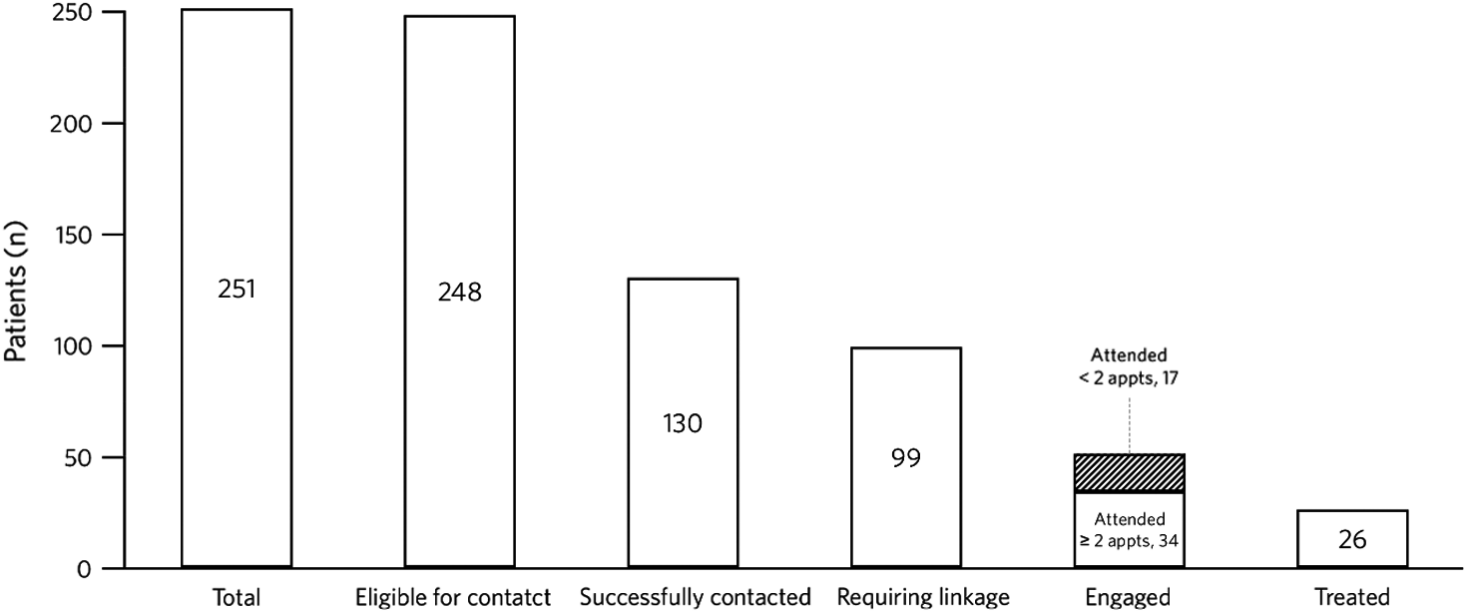
Linkage to care in patients with positive HCV‐Ag status. Abbreviations: appt, appointment; HCV‐Ag

Figure 3 describes the overall linkage to care cascade including the NHS Find and Treat programme subset (described below). Of the 251 patients who tested positive for HCV‐Ag, 3/251 (1.2%) died before they could be contacted. Of those alive, 130/248 (52%) were successfully contacted, of whom a further 12/130 (9%) died soon after being contacted. The cause of death was cancer not related to liver (six patients), decompensated liver disease (four patients) and multiple comorbidities (two patients). Of those successfully contacted, 19/130 (15%) were known and already engaged and 99/130 (76%) required linkage to care (new diagnosis or known but not engaged at time of test). Of those requiring linkage, 51/99 (52%) were linked locally (of these 50% were newly diagnosed), 5/99 (5%) were directed to alternative services as they did not reside locally and 41/99 (41%) declined an appointment at time of contact. Of those who were linked locally, 34/51 (66%) attended more than one appointment and, of these, 26/34 (76%) were successfully treated.

### 3.3 NHS Find and Treat subset (these numbers are included in the above results)

The NHS Find and Treat team were given a list of 87 patients with no fixed abode, and therefore, likely homeless, with an HCV‐Ag‐positive test result who the hospital study team had been unable to contact. Of those, 31/87 (35%) were successfully contacted, of whom 28/31 (90%) were eligible for care. Of those eligible for care, 10/28 (36%) attended the clinic and engaged with care and 19/28 (67%) were approved for treatment or started treatment.

## 4 DISCUSSION

### 4.1 Overall summary

To our knowledge, this 11‐month service evaluation of the real‐world sustainability of ED EPR opt‐out HCV and HBV testing is the largest intervention of its kind in the UK to date. It showed that opt‐out ED testing is practical in a large urban hospital setting and allows identification and linkage to care of significant numbers of active HBV and HCV infected individuals from marginalized groups that have historically had poor access to healthcare. In the current study, seroprevalence for HBsAg was 0.9% and for HCV‐Ag was 0.9%. The strata specific results mirror those seen in our previous 6‐week pilot study, showing the high burden of disease in central London.^21^ The demographic characteristics described here are in line with those reported previously in similar studies in UK.^25^, ^26^

It is noteworthy that prevalence of HCV‐Ag among patients aged 50–69 years was significantly higher than among those aged 16–49 years. This highlights a potential benefit of ED opt‐out testing: it captures the older patients who may no longer be attending services for people who inject drugs. Unfortunately history of drug use was not documented systematically in patients' notes and, therefore, formal analysis on this variable was not carried out.

Seroprevalence of both HCV‐Ab and HCV‐Ag appears to decrease over time (Figure S2). This could be affected by several factors including seasonal variation in attendance patterns and/or be an artefact of EPR testing: the system avoided repeat testing within 6 months of a positive result. We, therefore, feel any interpretation would be speculative.

Interestingly, HBsAg prevalence in the current study was almost twice as high as in the pilot, reasons for this are unknown but might be chance‐related due to the smaller sample size in the pilot phase. In the current study, 141/235 patients (60%) who were positive for HBsAg were men which might suggest that ED testing could be of particular benefit in identifying men with HBV, whereas many HBV infections in women are detected in prenatal screening.^25^, ^26^

Importantly, opt‐out ED testing provides an opportunity not only to identify new infections but also to re‐engage patients previously diagnosed but not linked to care, the latter accounting for around a quarter of HBV and half of HCV patients requiring linkage to care in our sample.

### 4.2 Testing in the ED

The testing uptake in the current study was 75% for both HBsAg and HCV‐Ab over the 11‐month evaluation period, demonstrating that EPR modification to create an automatic BBV order set and opt‐out testing achieved consistently higher uptake compared with more traditional testing approaches.^17^, ^18^, ^19^ HCV‐Ag testing allows identification of current HCV infection and is standard of care throughout our hospital. HCV‐Ag testing is cheaper than RNA‐based testing, is less complex for the laboratory and has a more rapid turnaround time.^27^ A linkage to care pathway based on such testing allows rapid focusing of resources on active cases, but does require recognition/safety‐netting to ensure HCV‐Ag‐negative cases are not excluded where relevant (as described in Methods).

For HCV testing, same‐sample (reflex) antigen tests were carried out in line with current European Association for the Study of the Liver guidelines.^28^ Reflex testing is essential for opportunistic screening programmes as it reduces the requirement for antibody‐positive patients to return for confirmatory blood draw, thus allowing resource focus on active infections.^19^

In contrast to usual practice, linkage to care was not the responsibility of the ED clinician who ordered the test, but rather that of the care coordinator. This takes out a significant barrier to ED clinical buy‐in and is reflected in guidance from the Royal College of Emergency Medicine, which states that ‘Safeguards are required before introducing routine Emergency Department HIV or blood‐borne virus testing. These safeguards include: a systems‐wide approach; adequate resources for training and education of staff, testing and follow‐up; and the development of robust protocols for the transfer of patient care with reactive or positive results to appropriate care and support services’.^29^

### 4.3 Linkage to care

While high levels of linkage to care were maintained for patients positive for HBV in the current study compared with the pilot study (87% and 93%, respectively), this was more challenging for HCV‐Ag‐positive patients in routine clinical practice (52% and 78%, respectively).^21^ It is noteworthy that 41% of HCV‐Ag‐positive patients contacted declined an appointment. However, among patients positive for HCV‐Ag, 26% of those who required linkage to care were successfully treated.

The difference in linkage to care between HBV and HCV cases very likely reflects differences in the patient populations. In accordance with previous studies^6^ showing that HCV disproportionately affects very marginalized populations (in particular people who inject drugs [PWIDs]), we found that homelessness was the most significant risk factor for HCV infection. Although not analysed formally, for the subset of patients who tested positive for HCV‐Ag and attended a clinic, it appeared that current intravenous drug users were more difficult to re‐engage than those with a history of intravenous drug use. Patients most likely to engage were those with fixed abode and those of older age.

In contrast, individuals with risk factors for HBV such as migration from countries with high HBV prevalence, and sexual contact with infected individuals^30^ likely experience less lifestyle‐related challenges to health seeking behaviour once diagnosed, also reflected in differential proportions for HBV and HCV having previously disengaged from care.

Largely, on account of these challenging patient populations, the team faced many barriers to linkage to care, especially in homeless patients, who frequently did not have an active mobile telephone number and/or were not registered with a primary care provider. System challenges to patient contact included data fragmentation and communication gaps between organizations, as well as the challenge of governance issues around confidentiality and information‐sharing rules.

Linkage to care approaches evolved throughout the service evaluation: at the start of the project, patient contact was consultant‐led, and later, led by a dedicated research nurse. For patients who could not be contacted despite previous efforts, their information was shared with hospital and regional community homeless teams. Especially for HCV, any linkage to care pathway needs to embrace primary, secondary and social care services.

A key component to the success of the NHS Find and Treat team was the use of ‘peer support’, which refers to involvement of people with lived experience of a lifestyle or condition and who share similar experiences or characteristics with the patient group being targeted.^31^ Community‐based peer‐support has been shown to improve linkage to care for HCV in homeless individuals and other high‐risk groups.^31^, ^32^ The local homeless teams and the NHS Find and Treat team used information from homeless databases and links with local homeless services to identify patients. Peer support workers with experience of homelessness went into the community to engage with patients. They accompanied patients to appointments, provided incentives and supported them through treatment.

### 4.4 Application in other settings

Routine, opt‐out BBV testing has been shown to be feasible in multiple hospitals serving large UK cities.^17^, ^18^, ^19^, ^20^, ^21^, ^22^ A service of this kind is likely to be most useful in urban areas, which often have populations with high incidence and prevalence of BBVs. We believe that, while this kind of testing could be implemented in almost any ED with an EPR system amenable to modification to include automatic requests, the integration of a linkage to care pathway requires good coordination between ED, clinical treatment and community teams. EPR modification can be applied to in‐patient and out‐patient settings as well, but EDs have the advantage of being accessible to all and, therefore, provide an unfiltered population.

### 4.5 Cost‐effectiveness

A previous initial economic evaluation using pilot study data suggested that ED testing for HBV and HCV in the UK is likely to be cost‐effective at a viral prevalence of 0.5% and above.^33^ The prevalence for both infections observed in the current study are well above this threshold. Ongoing work, using data from this and other large‐scale sustainability evaluations is looking at estimating the cost‐effectiveness of ED testing under real‐world conditions.

## 5 CONCLUSION

Our results demonstrate the effectiveness and sustainability of universal urban ED opt‐out HBV and HCV testing combined with integrated linkage to care pathways embracing secondary, primary and community care. Utilization of an EPR supported testing approach achieved sustainable high test uptake and our comprehensive linkage network better connecting existing services allowed care provision to traditionally poorly served, at‐risk groups that would not otherwise be possible without a large increase in health infrastructure.

## CONFLICT OF INTEREST

Gaia Nebbia has no conflict to declare. Murad Ruf is an employee of Gilead Sciences Ltd. Laura Hunter has have received travel grants and research grants from Gilead Sooria Balasegaram has no conflicts to declare. Terry Wong has served on advisory board meetings and as a paid speaker for Gilead, MSD, BMS and Norvartis. Ranjababu Kulasegaram has served as a speaker, consultant and advisory board member for ViiV Healthcare, Gilead Sciences and Merck Sharp & Dohme. Julian Surey has no conflicts to declare. Zana Khan has no conflicts to declare. Jack Williams has received a research grant funded by Gilead. Basel Karo has no conflicts to declare. Luke Snell has no conflicts to declare. Barnaby Flower received a travel grant from Gilead Sciences to attend a conference in 2017. Hannah Evans has no conflicts to declare. Sam Douthwaite has received travel grants from Gilead Sciences.

## AUTHOR CONTRIBUTIONS

All authors have made substantial contributions to conception and design, or acquisition of data, or analysis and interpretation of data; have been involved in drafting the manuscript or revising it critically for important intellectual content; and have given final approval of the version to be published. Each author has participated sufficiently in the work to take public responsibility for appropriate portions of the content and agree to be accountable for all aspects of the work in ensuring that questions related to the accuracy or integrity of any part of the work are appropriately investigated and resolved.

## Supporting information

Supplementary Material

## Data Availability

The data that support the findings of this study are available upon request from the corresponding author. The data are not publicly available due to privacy or ethical restrictions.
