# Supplementary material for: VirA+EmiC project: Evaluating real‐world effectiveness and sustainability of integrated routine opportunistic hepatitis B and C testing in a large urban emergency department
Source: J Viral Hepat. 2022 Apr 9;29(7):559–68. doi: 10.1111/jvh.13676 (PMC9322278; doi:10.1111/jvh.13676)
Supplement: Supplementary file 1 — Supplementary Material [file JVH-29-559-s001.docx]

**Supplementary information**

| **SUPPLEMENTARY TABLE 1** Univariable and multivariable Poisson regression models for demographic factors associated with positive HBsAg | | | | | | |
| --- | --- | --- | --- | --- | --- | --- |
| **Independent variables** | **Univariable model** | | | **Multivariable model^†^** | | |
|  | **PR** | **95% CI** | ***P* value** | **PR** | **95% CI** | ***P* value** |
| **Sex**  Female  Male | 1  1.5 | 1.2–2.0 | <0.001 | 1  1.7 | 1.3–2.2 | <0.001 |
| **Age group, years**  16–29  30–49  50–69  70+ | 1  3.4  4.0  1.0 | 2.2–5.3  2.5–6.3  0.5–2.0 | <0.001  <0.001  0.95 | 1  3.1  4.2  1.3 | 1.9–5.0  2.6–6.8  0.7–2.6 | <0.001  <0.001  0.42 |
| **Ethnicity**  White British  White other  Black/Black British  Asian  Mixed/other  Unknown | 1  6.6  17.4  17.2  7.3  7.7 | 3.4–12.5  9.6–31.6  8.9–33.1  3.5–15.5  4.0–14.9 | <0.001  <0.001  <0.001  <0.001  <0.001 | 1  6.8  17.5  16.1  7.6  6.9 | 3.6–13.0  9.7–31.5  8.3–31.3  3.6–16.1  3.5–13.5 | <0.001  <0.001  <0.001  <0.001  <0.001 |
| **Homeless**  No  Yes | 1  1.8 | 1.0–3.5 | 0.06 | 1  1.8 | 1.0–3.4 | 0.07 |
| **HIV status**  Negative  Positive  Unknown | 1  6.0  1.4 | 2.9–12.8  1.1–1.8 | <0.001  <0.001 | 1  4.0  1.4 | 1.9–8.6  1.1–1.8 | <0.001  0.02 |
| **ED arrival day**  Weekday  Weekend | 1  0.9 | 0.7–1.3 | 0.69 | 1  1.1 | 0.8–1.5 | 0.65 |
| **ED arrival time**  Day (08:00±19:59)  Night (20:00±07:59) | 1  0.7 | 0.6–1.0 | 0.04 | 1  0.7 | 0.5–0.9 | 0.01 |
| ^†^Cases with available information for all predicting factors were included in the multivariable analysis (N=26,153/27,646).  Abbreviations: CI, confidence interval; ED, emergency department;HBsAg, hepatitis B surface antigen; HIV, human immunodeficiency virus; PR, prevalence ratio. | | | | | | |

| **SUPPLEMENTARY TABLE 2** Multivariable Poisson regression models for factors associated with positive HCV-Ab (model 1) and HCV-Ag (model 2) | | | | | | |
| --- | --- | --- | --- | --- | --- | --- |
| **Independent variables** | **Model 1^†^**  **HCV-Ab** | | | **Model 2^†^**  **HCV-Ag** | | |
|  | **IRR** | **95% CI** | ***P* value** | **IRR** | **95% CI** | ***P* value** |
| **Sex**  Female  Male | 1  2.0 | 1.6–2.5 | <0.01 | 1  2.5 | 1.7–3.5 | <0.01 |
| **Age group, years**  16–29  30–49  50–69  70+ | 1  4.3  4.0  1.2 | 3.0–6.2  2.8–5.9  0.7–2.0 | <0.01  <0.01  0.52 | 1  3.6  3.2  0.9 | 2.2–6.0  1.9–5.4  0.4–2.0 | <0.01  <0.01  0.93 |
| **Ethnicity**  White British  White other  Black/Black British  Asian  Mixed/other  Unknown | 1  0.7  0.4  0.4  0.6  0.6 | 0.5–0.9  0.3–0.5  0.2–0.6  0.4–0.9  0.5–0.8 | <0.01  <0.01  <0.01  <0.01  <0.01 | 1  0.6  0.4  0.3  0.5  0.4 | 0.4–0.9  0.2–0.6  0.1–0.8  0.3–0.9  0.3–0.7 | 0.01  <0.01  0.02  0.02  <0.01 |
| **Homeless**  No  Yes | 1  10.7 | 8.7–13.1 | <0.01 | 1  16.6 | 12.5–22.1 | <0.01 |
| **HIV status**  Negative  Positive  Unknown | 1  4.3  1.2 | 2.7–6.8  1.0–1.5 | <0.01  0.04 | 1  2.8  0.8 | 1.5–5.1  0.7–1.1 | <0.01  0.24 |
| **ED arrival day**  Weekday  Weekend | 1  0.9 | 0.7–1.1 | 0.17 | 1  1.0 | 0.8–1.3 | 0.92 |
| **ED arrival time**  Day (08:00±19:59)  Night (20:00±07:59) | 1  1.1 | 0.9–1.3 | 0.21 | 1  1.1 | 0.8–1.4 | 0.63 |
| ^†^Cases with available information for all predicting factors were included in the multivariable analysis (model 1: N=26,164 and model 2: N=26,104). In both models, parameter estimates were obtained using robust standard errors to control for mild violation of underlying assumptions.  Abbreviations: CI, confidence interval; ED, emergency department; HCV-Ab, hepatitis C antibody; HCV-Ag, hepatitis C antigen; HIV, human immunodeficiency virus; IRR, incidence rate ratio. | | | | | | |

**SUPPLEMENTARY FIGURE 1.** Patients with opt-out hepatitis B testing and seroprevalence of HBsAg (A) during the study period (phases 1 and 2), (B) by age groups

A


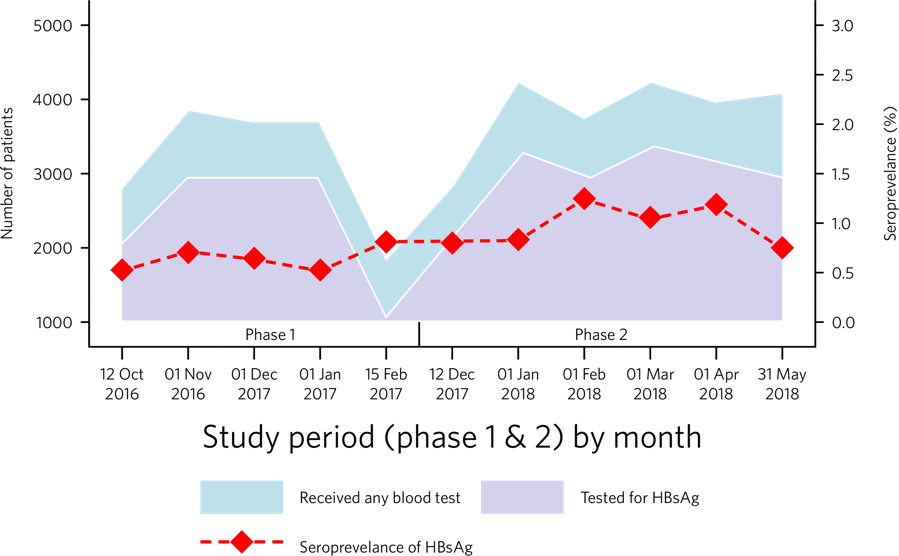


B


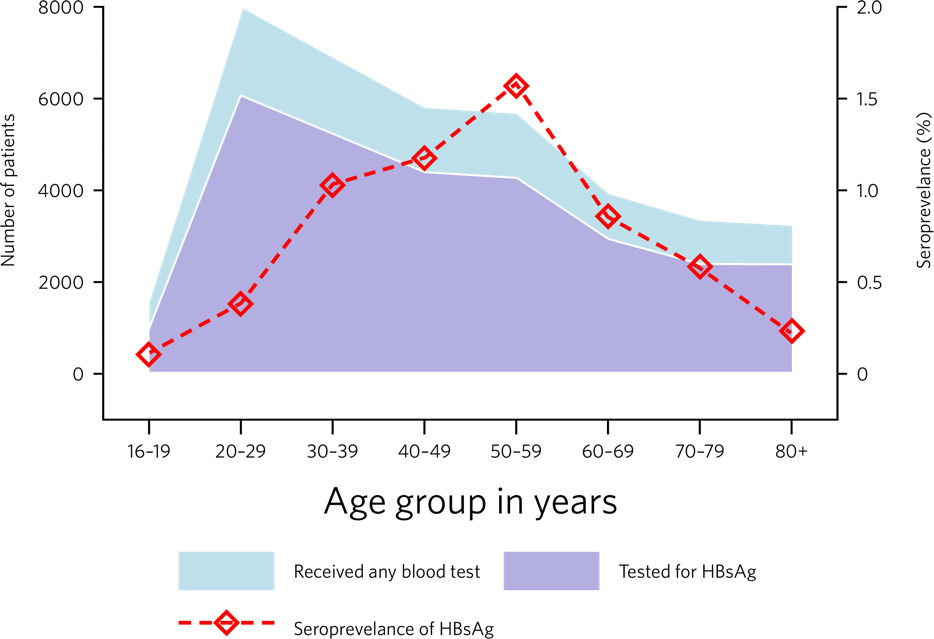


Abbreviation: HBsAg, hepatitis B surface antigen.

**SUPPLEMENTARY FIGURE 2.** Patients with opt-out hepatitis C testing and seroprevalence of HCV-Ab and HCV-Ag (A) during the study period phases 1 and 2), (B) by age groups

A


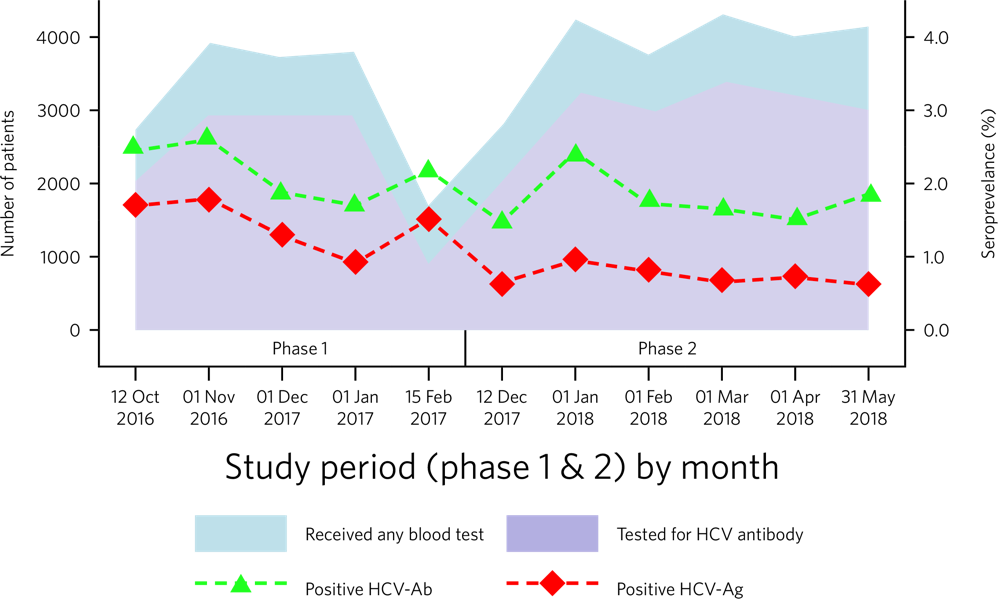


B

**
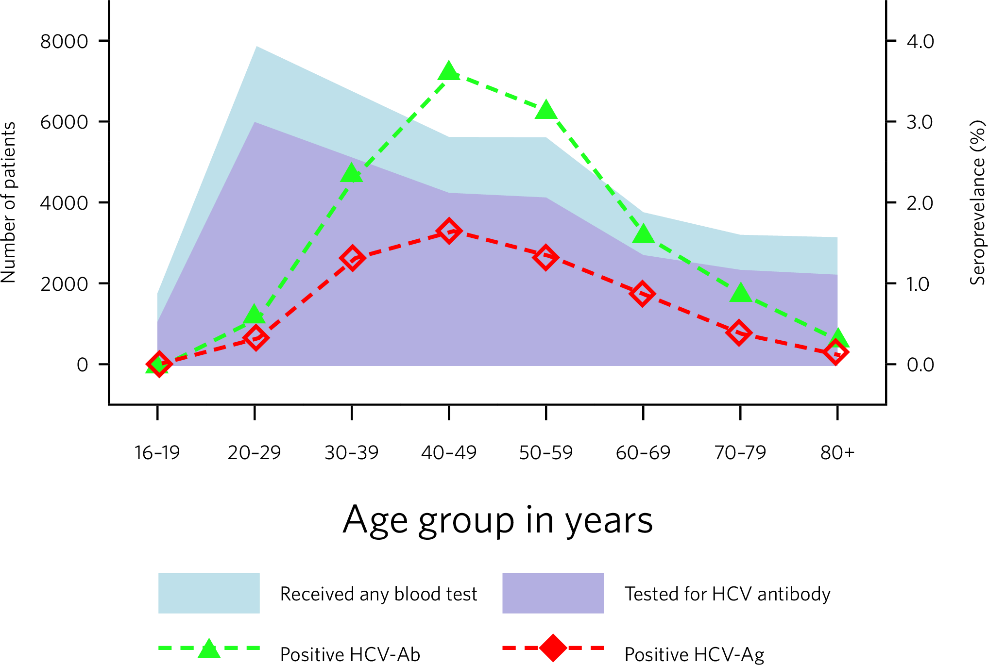
**

Abbreviations: HCV-Ab, hepatitis C virus antibody; HCV-Ag, hepatitis C virus antigen
